# Neoadjuvant Programmed Cell Death 1 (PD-1) Inhibitor Treatment in Patients With Hepatocellular Carcinoma Before Liver Transplant: A Cohort Study and Literature Review

**DOI:** 10.3389/fimmu.2021.653437

**Published:** 2021-07-19

**Authors:** Zi-yun Qiao, Zi-jie Zhang, Zi-cheng Lv, Huan Tong, Zhi-feng Xi, Hao-xiang Wu, Xiao-song Chen, Lei Xia, Hao Feng, Jian-jun Zhang, Qiang Xia

**Affiliations:** ^1^ Department of Liver Surgery, Renji Hospital, School of Medicine, Shanghai Jiao Tong University, Shanghai, China; ^2^ Clinical and Translational Research Center, Shanghai First Maternity and Infant Hospital, Shanghai, China; ^3^ Shanghai Engineering Research Centre of Transplantation and Immunology, Shanghai, China; ^4^ Shanghai Institute of Transplantation, Shanghai, China

**Keywords:** programmed cell death 1 (PD-1) inhibitor, liver transplant, hepatocellular carcinoma, allograft rejection, neoadjuvant immunotherapy

## Abstract

Programmed cell death 1 (PD-1) blockade is considered contraindicated in liver transplant (LT) recipients due to potentially lethal consequences of graft rejection and loss. Though post-transplant PD-1 blockade had already been reported, pre-transplant use of PD-1 blockade has not been thoroughly investigated. This study explores the safety and efficacy of neoadjuvant PD-1 blockade in patients with hepatocellular carcinoma (HCC) after registration on the waiting list. Seven transplant recipients who underwent neoadjuvant PD-1 blockade combined with lenvatinib and subsequent LT were evaluated. The objective response rate (ORR) and disease control rate (DCR) was 71% and 85% according to the mRECIST criteria. Additionally, a literature review contained 29 patients were conducted to summarize the PD-1 blockade in LT for HCC. Twenty-two LT recipients used PD-1 inhibitors for recurrent HCC. 9.1% (2/22) and 4.5% (1/22) recipients achieved complete remission (CR) and partial remission (PR), respectively; 40.9% (9/22) recipients had progressive disease (PD). Allograft rejection occurred in 45% of patients. In total, seven patients from our center and three from the literature used pretransplant anti-PD-1 antibodies, eight patients (80%) had a PR, and the disease control rate was 100%. Biopsy-proven acute rejection (BPAR) incidence was 30% (3 in 10 patients), two patients died because of BPAR. This indicated that neoadjuvant PD-1-targeted immunotherapy plus tyrosine kinase inhibitors (TKI) exhibited promising efficacy with tolerable mortality in transplant recipients under close clinical monitoring.

## Introduction

Immune checkpoint inhibitors (ICIs) have achieved remarkable results in the treatment of several kinds of cancers (1). The immune checkpoint proteins cytotoxic T-lymphocyte- associated-4 (CTLA-4) and programmed cell death protein 1 (PD-1) are receptors expressed on the surface of cytotoxic T cells, whose ligands are CD80/CD86 and programmed death-ligand 1 (PD-L1), respectively. The two pathways downregulate T-cell activation to maintain peripheral tolerance and help cancer cells to escape from cytotoxic T cell-mediated death (2). ICIs are inhibitors of immune checkpoints. They prevent receptors and ligands from engagement, thus playing an antitumor effect (3).

Various studies showed potential antitumor activities and a manageable safety profile in ICIs for HCC treatment. However, due to concerns about postoperative fatal rejection, ICIs were rarely included in treating patients receiving solid organ transplants (4). There were still a small number of solid organ transplantation patients, including liver transplant (LT) recipients who received immunotherapy postoperatively to fight against cancer recurrence and *de novo* malignancy.

Concerning neoadjuvant immunotherapy, it has been revived in the past couple of years, with emerging data from several ongoing trials suggesting that neoadjuvant immunotherapy may have significant efficacy and could potentially improve the survival of patients with different solid tumors. Though neoadjuvant immunotherapy with the PD-1 inhibitor is sufficient prior to surgery for early solid cancers, its safety and feasibility in LT were far from being explored. In July 2020, the authors started a randomized control trial (NCT04425226) to evaluate the safety and efficacy of PD-1 inhibitor in combination with lenvatinib as neoadjuvant therapy in participants with hepatocellular carcinoma (HCC) before LT. Before the trial, a retrospective cohort study was conducted. This article retrospectively investigated seven LT recipients who underwent a liver transplant after using pembrolizumab or camrelizumab in our center and summarized the reported cases that used PD-1 inhibitors plus tyrosine kinase inhibitors (TKI) before or after liver transplantation.

## Methods

### Patients and Clinical Characteristics

A retrospective cohort study was conducted. Seven LT recipients who underwent neoadjuvant pembrolizumab (Keytruda, 200mg, 3 weeks per cycle) or camrelizumab (AiRuiKa™, 200mg, 2 weeks per cycle) combined with lenvatinib (LENVIMA^®^) before LT in 2020 and 2021 from Renji Hospital were recruited. Additionally, a literature review contained a total of twenty-nine patients were conducted to summarize the PD1 treatment in LT.

### Peri-Transplant Management and Immunosuppression

Chest computed tomography (CT), abdominal contrast-enhanced CT scan with three-dimensional angiography, and enhanced magnetic resonance imaging (MRI) were routinely performed. High suspicion of distant tumor metastasis explored by imaging were contraindications for LT. Liver transplants at Shanghai Renji Hospital are conducted using standard techniques without the use of venovenous bypass. All patients were admitted to the transplant ICU immediately after LT. Our institution’s immunosuppression regimen includes early postoperative use of basiliximab induction and a tapered dose of corticosteroids, and long-term maintenance with combination or separate use of corticosteroids, a calcineurin inhibitor (cyclosporine or tacrolimus), sirolimus, and mycophenolate mofetil depending on the patient’s condition. After discharge, the patients were followed at the outpatient clinic according to the standard protocol of our center (5).

### Flow Cytometry and Intracellular Cytokine Assays

Relative proportions and number of CD4+CD3+T cell, CD8+CD3+T cell, Treg, and NK cell subsets were analyzed by flow cytometry using a FACS CANTO II (BD) instrument and FlowJo software (Tree Star). IL-2, IL-6, IL-8, IL-10, IL-12p, IL-17, TNF-α, IFN-α, and IFN-γ, were also analyzed by flow cytometry by the clinical laboratory.

### Characteristics of the Included Studies

A total of 13 potential articles were collected from PubMed with the searching strategy: ((((((PD1[Title/Abstract]) OR (PD-1[Title/Abstract])) OR (programmed cell death 1[Title/Abstract])) OR (checkpoint inhibitor[Title/Abstract])) OR (ICI[Title/Abstract])) AND (liver transplant[Title/Abstract])) AND ((hepatocellular carcinoma[Title/Abstract]) OR liver cancer[Title/Abstract]). Articles from the ISI Web of Science, EMBASE, and the Cochrane Library were also uploaded. Studies of other diseases, studies that were not related to TILs, studies of mouse models or cell lines, review articles, and duplicate publications were excluded after reviewing the titles and abstracts. A total of 16 articles, including 29 patients, remained for full-text review.

## Results

In our study, all seven patients started to use camrelizumab or pembrolizumab combined with lenvatinib because of HCC and received LT after downstage treatments. One patient experienced graft rejection after LT, but after adjusting the immunosuppression regimen, his liver function returned to normal.

### Patients’ Clinical Characteristics in Three Cohorts

A total of 7 patients using neoadjuvant PD-1 inhibitor in Renji Hospital were included in cohort1, patients using neoadjuvant or adjuvant PD-1 inhibitor in the literature were included in cohort2 and cohort3 (**Table 1**). In cohort1, the washout period was set as 42 days, shorter than cohort2, which included three literature-reported neoadjuvant PD1 treated patients. However, the incidence of Biopsy-proven acute rejection (BPAR) was 14.3%, less than cohort2 and cohort3, which were 67% and 23.1%, respectively. Neoadjuvant PD-1 blockade exhibited promising partial remission (PR), which were 71% and 67% in cohort1 and 2, respectively, compared to post-transplant PD-1 blockade. Neoadjuvant PD-1 blockade in our center also showed a lower BPAR rate.

**Table 1:** Comparison of patients’ clinical characteristics in three cohorts.

	Cohort1 (Neoadjuvant PD1 treatment RJ)	Cohort2 (Literature-neoadjuvant PD1 treatment)	Cohort3 (Literature-post-LT PD1 treatment)
**M/F**	7/0	3/0	20/6
**Age**	53 ± 12.1	62 ± 14.2	60 ± 15.3
**Cycles**	3(1-5)	29(10-44)	6(1-27)
**Time between LT and ICI(months)**	1.3	29.0	42.4
**PR%**	71%	67%	5%
**CR%**	0%	0%	10%
**PD%**	0%	0%	43%
**BPAR%**	14.3%	67%	23.1%

RJ, Renji Hospital; PR, partial remission; CR, complete remission; PD, progressive disease; LT, liver transplantation; BPAR, Biopsy-proven acute rejection.

### Post-Transplant Liver Function Recovery

**Figure 1:**
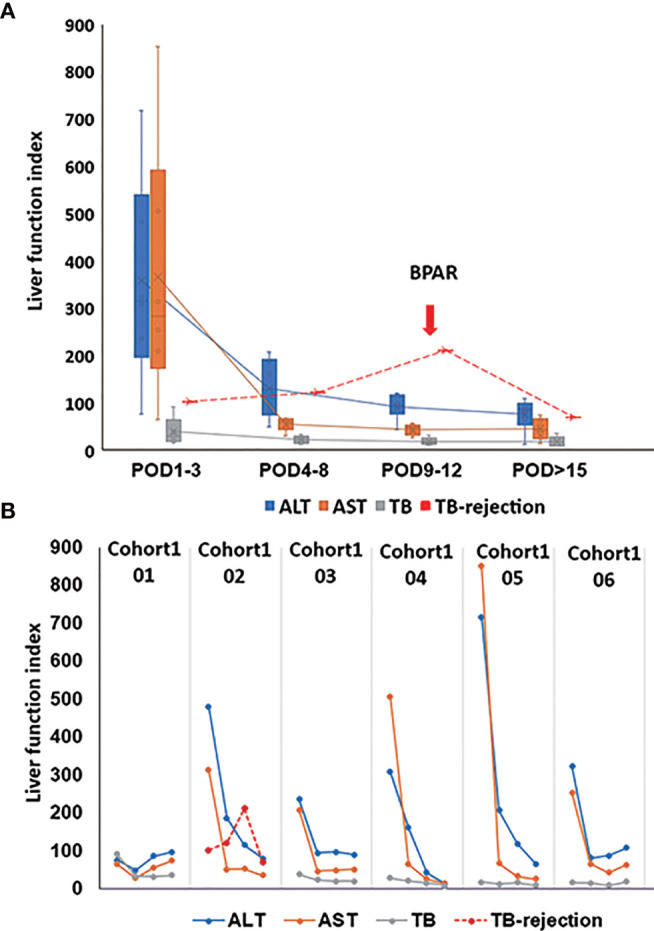
Post-transplant liver function. **(A)** The change of average ALT, AST, and TB from POD1 to POD>15. The red line of TB rejection presents the change of TB for the patient with BPAR; BPAR occurred in the combination of high TB. **(B)** The presentative liver function from six out of seven patients in cohort1. For patient02 in cohort1, his transaminase increased with a peak AST level at 785 U/L on POD1 and gradually decreased. However, his TB level continued to grow after the surgery. On POD 10, his TB reached 309.8 μmol/L. As a possibility of BPAR was suspected, he underwent a puncture liver biopsy on POD 11. The pathological results suggested a mild acute rejection (rejection activity index, RAI=2+1+2 = 5). Simultaneously, a dose of 500 mg of methylprednisolone was given from POD 11, then tapered down and discontinued on POD 16. The patient’s TB level fell below 100 μmol/L and did not increase subsequently. ALT, alanine aminotransferase; AST, aspartate aminotransferase; TB, total bilirubin; biopsy-proven acute rejection (BPAR).

The postoperative immunosuppressive treatments were presented in **Supplementary Figure 1**, respectively. 85.7% of the patients’ liver function recovered quickly after the surgery. The peaks of transaminase and total bilirubin appeared on POD 1, then gradually dropped to the normal range during the follow-up examination. Creatinine remained within the normal range during hospitalization (**Figure 1A**). Nevertheless, the patient02 in cohort1 (**Figure 1B**) suffered from mild acute rejection. On POD 1, his transaminase increased, with a peak AST level at 785 U/L, and then gradually decreased. However, his total bilirubin level continued to grow after the surgery. On POD 10, the patient’s total bilirubin reached 309.8 μmol/L. As a possibility of BPAR was suspected, he underwent a puncture liver biopsy on POD 11. The pathological results suggested a mild acute rejection (rejection activity index, RAI=2+1+2 = 5) (**Supplementary Figure 2**). Simultaneously, a dose of 500 mg of methylprednisolone was given from POD 11, then tapered down and discontinued on POD 16. The patient’s total bilirubin level fell below 100 μmol/L and did not increase subsequently. He was re-admitted to the hospital at POD 44 due to a continuous hemoglobin decrease (41g/L). His liver function remained stable, but the examination showed a Cytomegalovirus (CMV) infection (520copies/mL). There was no evidence of other disorders.

### Alteration of the Proportion of Immune Cells and Cytokines

**Figure 2:**
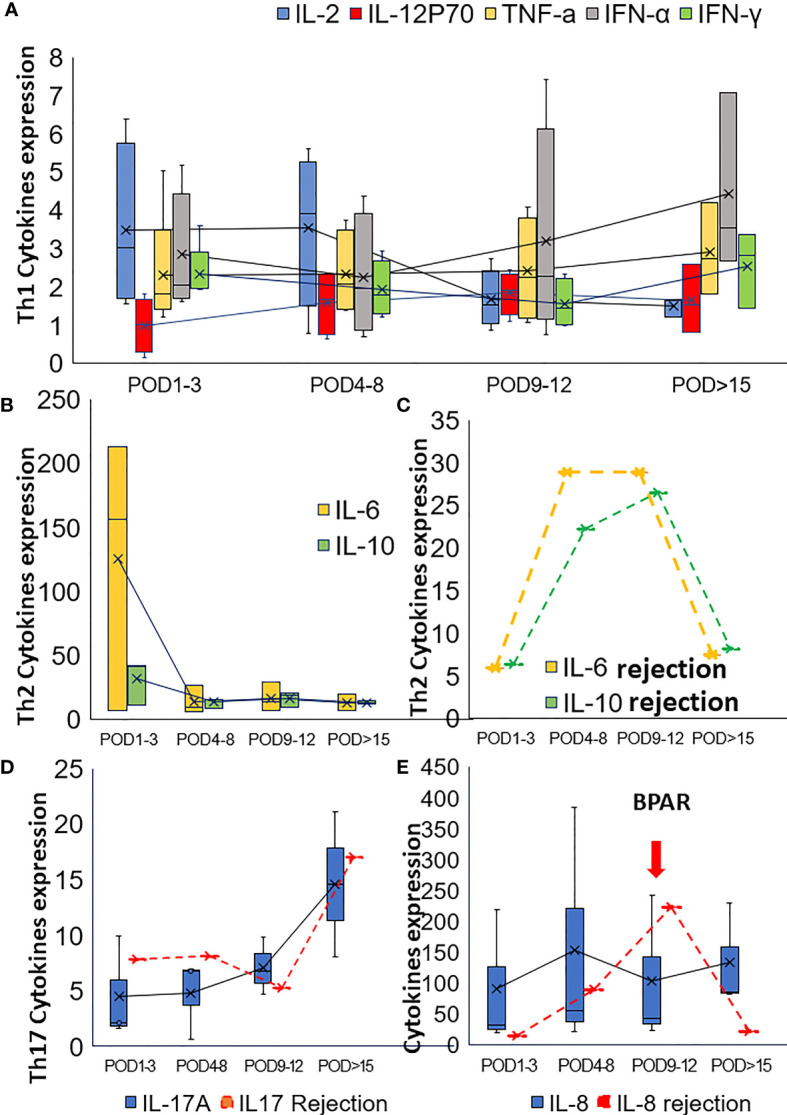
Alteration of the cytokines in post-transplant set-up. **(A)** Alteration of Th1 cytokines in the post-transplant set-up; **(B)** The change of Th2 cytokines after transplantation for patients with neoadjuvant PD-1 blockade. **(C)** Alteration of Th2 cytokines (IL6 and IL10) for the patient with BPAR in cohort1. The alteration of Th17 cytokine IL17 **(D)** and IL8 **(E)** were also presented in this figure.

To reveal the effect of pretransplant PD-1 blockade in the post-transplant situation, the soluble cytokines were also examined. In cohort1, concerning Th1-type cytokines, such as IL2, IFN-γ, TNFα, and IL12p70, IL2 decreased gradually, and IFN-α increased slightly. TNFα and IL12p70 were restored steadily after the operation(**Figure 2A**). IL2 and IFN-γ increased rapidly from POD1 to POD5 and POD7, respectively, for the third patient who got subsequent allograft rejection.

**Figure 3:**
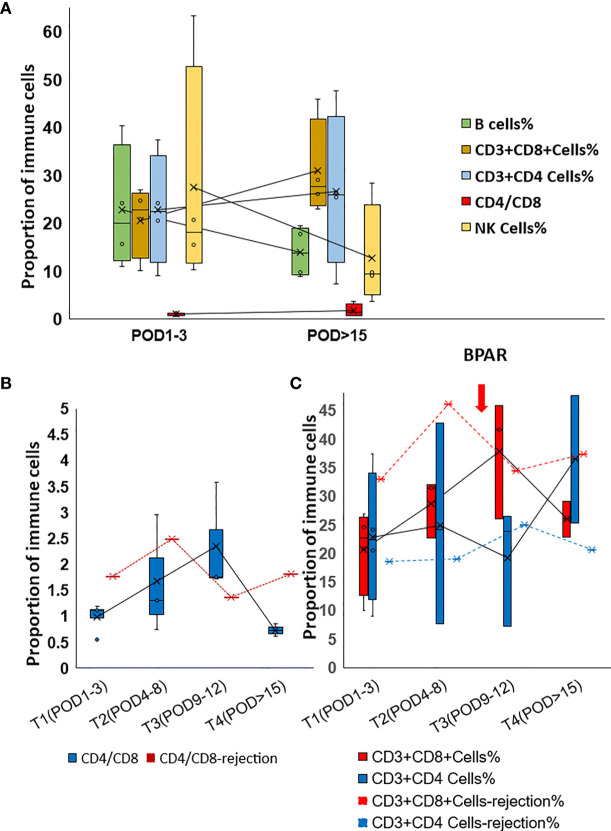
Alteration of the proportion of Immune cells in post-transplant set-up. **(A)** Changes of lymphocyte percentages and CD4/CD8 ratio in cohort 1 patients. **(B)** Changes of CD4/CD8 ratio in rejection and non-rejection patients. **(C)** Changes of lymphocyte percentage in rejection(red dot line and blue dot line) and non-rejection patients.

Th2-type cytokines, especially IL6 and IL10, gradually dropped to the normal range during the follow-up examination (**Figure 2B**). However, increased IL6 and IL10 were detected from POD5 to POD11 (**Figure 2C**). Generally, increased IL-17 was observed in the present study. However, IL17 slightly dropped while IL8 increased when allograft rejection occurred (**Figures 2D, E**). The proportion of peripheral lymphocytes was analyzed (**Figures 3A–C**), decreased NK cells and B cells, and increased CD3+CD8+cells were found in cohort1. A rapid increase of CD4/CD8 ratio and CD8+CD3+ T cells were found for the patient with post-transplant allograft rejection from POD1 to POD5(**Figures 3B, C**).

### Literature Review and Analysis

There have been several case reports in the literature of PD-1/CTLA-4 inhibitor treatment in transplant recipients. In total, 29 patients were included in the analysis (6–20). Twenty-two LT recipients used PD-1 inhibitors; one patient used CTLA-4 inhibitors for HCC. Five patients used PD-1 inhibitors for different types of *de novo* malignancies: 2 for melanoma, 1 for colon adenocarcinoma, 1 for malignant peripheral nerve sheath tumor (MPNST), and 1 for non-melanoma skin cancer. The other patient used PD-1 inhibitors for recurrent ICC (**Table 2**).

**Table 2:** Case reports on the use of ICI in LT patients.

Author	Sex	Age	Tumor type	de novo/	ICI	Duration	Pre/PostLT	Time between LT and ICI	Immunosuppression drugs	Outcome	Rejection
*Chen. 2021*	*M*	*39*	*HCC*	*recurrent*	*toripalimab*	*10 cycles*	*before*	*93days*	*tacrolimus, steroids*	*N/A*	*Y*
*Al Jarroudi O. 2020*	*M*	*70*	*HCC*	*recurrent*	*nivolumab*	*4 cycles*	*after*	*3 years*	*tacrolimus*	*N/A*	*Y*
	*F*	*62*	*HCC*	*recurrent*	*nivolumab*	*5 cycles*	*after*	*2.5 years*	*tacrolimus*	*PD*	*N*
	*M*	*66*	*HCC*	*recurrent*	*nivolumab*	*6 cycles*	*after*	*5 years*	*tacrolimus*	*PD*	*N*
*Shi et al., 2020*	*M*	*59*	*ICC*	*recurrent*	*toripalimab*	*8 cycles*	*after*	*N/A*	*N/A*	*PD*	*N*
	*M*	*46*	*HCC*	*recurrent*	*toripalimab*	*7cycles*	*after*	*N/A*	*N/A*	*PD*	*N*
	*M*	*46*	*HCC*	*recurrent*	*toripalimab*	*3 cycles*	*after*	*N/A*	*N/A*	*SD*	*N*
	*M*	*62*	*HCC*	*recurrent*	*toripalimab*	*2 cycles*	*after*	*N/A*	*N/A*	*N/A*	*N*
	*M*	*66*	*HCC*	*recurrent*	*toripalimab*	*single use*	*after*	*N/A*	*N/A*	*N/A*	*N*
*Pandey et al., 2020*	*F*	*65*	*HCC*	*recurrent*	*ipilumumab*	*7 cycles*	*after*	*7.2 years*	*tacrolimus*	*CR*	*N*
*SchwachaEipper.2020*	*M*	*62*	*HCC*	*de novo*	*nivolumab*	*34 cycles*	*before*	*1.5 years*	*N/A*	*PR*	*N*
*Nordness et al.2020*	*M*	*65*	*HCC*	*de novo*	*nivolumab*	*44 cycles*	*before*	*1.8 years*	*tacrolimus, MMF, and prednisone*	*PR*	*Y*
*Amjad et al.2019*	*F*	*62*	*HCC*	*recurrent*	*nivolumab*	*20 months*	*after*	*1.3 years*	*tacrolimus, mycophenolate*	*CR*	*N*
*Lee et al.2019*	*M*	*73*	*NMSC*	*de novo*	*nivolumab*	*2 cycles*	*after*	*12 years*	*cyclosporine, everolimus*, *steroids, mycophenolate and sirolimus*	*N/A*	*Y, RAI=9*
*Chen et al.2019*	*M*	*61*	*CRC*	*de novo*	*pembrolizumab*	*15 cycles*	*after*	*3.5 years*	*tacrolimus, steroids, MMF*	*PR*	*N*
*Kuo et al., 2018*	*F*	*62*	*MPNST*	*de novo*	*pembrolizumab*	*25 cycles*	*after*	*6.3 years*	*tacrolimus, sirolimus, MMF*	*PR*	*N*
*Gassmann. 2018*	*F*	*53*	*HCC*	*recurrent*	*nivolumab*	*1 cycle*	*after*	*2 years*	*tacrolimus, steroids, MMF, everolimus*	*N/A*	*Y, RAI=7*
*DeLeon et al., 2018*	*M*	*56*	*HCC*	*recurrent*	*nivolumab*	*1.2 m*	*after*	*2.7 years*	*tacrolimus*	*PD*	*N*
	*M*	*54*	*Melanoma*	*de novo*	*pembrolizumab*	*9.5 m*	*after*	*5.5 years*	*MMF, everolimus*	*CR*	*N*
	*M*	*55*	*HCC*	*recurrent*	*nivolumab*	*1.1 m*	*after*	*7.8 years*	*MMF, sirolimus*	*PD*	*N*
	*F*	*34*	*HCC*	*recurrent*	*nivolumab*	*1.3 m*	*after*	*3.7 years*	*tacrolimus*	*PD*	*N*
	*M*	*63*	*HCC*	*recurrent*	*nivolumab*	*0.3 m*	*after*	*1.2 years*	*tacrolimus*	*N/A*	*N*
	*M*	*68*	*HCC*	*recurrent*	*nivolumab*	*0.9 m*	*after*	*1.1 years*	*sirolimus*	*N/A*	*Y*
	*M*	*63*	*Melanoma*	*de novo*	*pembrolizumab*	*1 cycle*	*after*	*3.1 years*	*MMF, steroids*	*N/A*	*Y*
*Ashwin et al., 2018*	*M*	*57*	*HCC*	*recurrent*	*pembrolizumab*	*10 m*	*after*	*4.3 years*	*tacrolimus, steroids, MMF, sirolimus*	*PR*	*N*
*Friend et al., 2017*	*M*	*20*	*HCC*	*recurrent*	*nivolumab*	*2 cycles*	*after*	*3 years*	*sirolimus*	*N/A*	*Y*
	*M*	*14*	*HCC*	*recurrent*	*nivolumab*	*2 cycles*	*after*	*3 years*	*tacrolimus*	*N/A*	*Y*
*De Toni et al., 2017*	*M*	*41*	*HCC*	*recurrent*	*nivolumab*	*15 cycles*	*after*	*11 months*	*tacrolimus*	*PD*	*Y*
*Varkaris et al., 2017*	*M*	*70*	*HCC*	*recurrent*	*pembrolizumab*	*3 months*	*after*	*8 years*	*tacrolimus*	*PD*	*N*

HCC, hepatocellular carcinoma; ICC, intrahepatic cholangiocarcinoma; NMSC, non-melanoma skin cancer; CRC, colon adenocarcinoma; MPNSC, malignant peripheral nerve sheath tumor; MMF, Mycophenolate mofetil; PD, progressive disease; SD, stable disease; CR, complete remission; PR, partial remission; RAI, rejection activity index.

Three patients used ICI before liver transplantation, and the remaining 26 used ICI after the surgery. 9.1% (2/22) LT recipients with HCC and one patient with *de novo* melanoma achieved complete remission (CR) (8, 10); 13.6% (3/22) patients with HCC (7, 9) and two with *de novo* malignancies (13, 14). had a partial remission (PR) after immunotherapy; 4.5% (1/22) patient with HCC remained stable disease (SD) (6); 40.9% (9/22) LT recipients with HCC and one patient with recurrent ICC had progressive disease (PD). The tumor status of 10 patients was unknown (N/A), some of which were not reported in the literature; the others could not be assessed due to the rapid emergence of severe rejection after medication.

Nivolumab was used as an immunotherapy regimen in 16 patients’ therapy; pembrolizumab was used in 6 cases; 6 patients used toripalimab; the other used ipilumumab. Of ten out of the twenty-nine liver transplant recipients who received ICI and developed with allograft rejection, two patients underwent ICI treatment before LT. The other eight started postoperatively.

Al Jarroudi O et al. reported a male patient who used salvage nivolumab for recurrent HCC after LT and stopped the therapy after four cycles because of elevated liver enzymes and total bilirubin (11). Nordness, Mina F., et al. reported a patient diagnosed with HCC and nivolumab significantly affected tumor burden reduction (9). He stopped nivolumab one week before liver transplantation, but his liver biopsy on POD 6 demonstrated acute hepatic necrosis with profound lymphocytic infiltration in the portal tracts and died on POD 10. Lee, B. T., et al. reported a patient with newly diagnosed non-melanoma skin cancer 12 years after LT due to HCC (12). Surgery and chemotherapy failed to achieve an effective outcome as the tumor recurrence, so he started treatment with nivolumab and changed the immunosuppressive drug from tacrolimus to cyclosporine in advance. After the second infusion, his liver function deteriorated rapidly, and a liver biopsy revealed a rejection activity index (RAI) of 9/9. Subsequently, the patient adjusted the immune transplantation protocol many times, but the clinical status did not improve, and he developed renal failure at the end of comprehensive treatment. The patient reported by Gassmann, D. et al. started nivolumab treatment due to the recurrence of HCC without any adjustments to the immunosuppressive regimen (15). Two weeks after administration, a liver biopsy revealed acute rejection with an RAI of 7. Since then, the patient’s condition deteriorated, with extensive liver necrosis and cerebral hemorrhage, accompanied by increased brain pressure. He eventually died 25 days after taking nivolumab for the first time. Among the seven patients reported by Deleon, there were 2 cases of rejection (16). One patient’s liver function improved under immunosuppressive treatment, and the other patient died due to rejection. Friend et al. reported two patients with recurrent and metastatic liver cancer after transplantation. Both experienced severe acute rejection within one month after starting nivolumab and died of fulminant hepatic failure because of rejection.

## Discussion

### ICIs Led to a Higher Risk of Allograft Rejection?

After an organ transplant, immune tolerance induction is a crucial part of the transplant’s survival. Both the PD-1 and CTLA-4 signaling pathways are closely related to immune tolerance after transplantation. Previous studies have shown that the PD-1/PDL-1 co-inhibitory pathway was influential in regulating graft tolerance and rejection, both in animal models and in humans (21). PD-L1 and PD-1 are abundantly expressed in liver allografts, and that PD-1/PD-L1 blockade leads to enhanced intragraft T cell proliferation to allostimulation, thus counter-regulating graft rejection after organ transplantation in humans. In liver allografts, PD-1 was expressed in infiltrating T cells, PD-L1 was expressed in hepatocytes, bile duct cells, and along the sinusoids. Blocking the PD-1/PD-L1 pathway leads to increased allogeneic stimulation T cells proliferate in the transplanted body, thereby participating in the counter-regulation of transplant rejection (22). Due to the lack of safety-related large-scale clinical trials, there were often hesitations in formulating immunotherapy for liver recipients with malignant tumors.

In our report and literature search, a total of 36 patients who underwent PD-1/CTLA-4 inhibitor therapy before or after LT were included. Allograft rejection occurred in 50% (8/16) of nivolumab, 33% (1/3) of camrelizumab, 17% (1/6) of toripalimab, and 17% (1/6) of pembrolizumab treated patients. 5 out of the ten patients died of fatal rejection, two patients died of tumor progression. The other three patients remained alive after the timely intervention. Though the incidence of BPAR was 67% in the literature for neoadjuvant PD-1 blockade, it was only 14.3% in our center. Based on the above data, we suppose that ICIs could achieve considerable safety under close clinical monitoring and could be applied to the preoperative down-stage of liver transplantation and the tumor treatment of transplant recipients.

### Immunological Biomarker for Predicting Rejection

We suggest that liver biopsies of the allografts are taken routinely before PD-1 treatment. The lack of BPAR during ipilimumab treatment reflects a predominant role of PD-1 in determining graft tolerance. Positive PD-L1 staining in biopsy suggest higher risk of BPAR, then initiation of therapy with a CTLA4-blocking agent could be considered. A clinical trial involving six liver transplant recipients, in which 1 with a positive PD-L1 expression and five without PD-L1 expression in their grafts, showed that none of the PD-L1 expression negative patients had rejection. In contrast, the positive patient suffered a graft rejection (6). This indicates that PD-L1 expression may be a marker of organ rejection in the anti-PD-1 treatment of liver transplant recipients.

Pretreatment with steroids can be attempted in the absence of obvious contraindications. The value of the CD4/CD8 ratio might also be helpful for rejection prediction. A lower CD4/CD8 ratio in the lymph node was proved to be related to a higher risk for acute rejection after kidney and lung transplantation (23, 24).

### Could Immunotherapy Achieve Considerable Antitumor Efficacy?

Immunotherapy for preoperative downstage and liver transplant recipients has achieved considerable results. Neoadjuvant PD-1 blockade exhibited promising partial remission (PR), 71% and 67% in cohort1 and 2, respectively, compared to post-transplant PD-1 blockade. This may indicate that ICIs had a positive therapeutic effect on transplant recipients.

### The Interval Between the PD-1 Inhibitor Treatments and Transplant

It is currently believed that the application of immunotherapy within a short period before or after transplantation is more likely to produce fatal rejection. Schwacha-Eipper et al. believed that nivolumab should be stopped six weeks before transplantation, considering its 4-week half-life (7). In our patients, the last application of camrelizumab was 3-8 weeks before liver transplantation. Although the patient treated with camrelizumab three weeks before surgery did not experience adverse reactions during his hospitalization, the timing of ICIs needs to be further explored. In one ongoing trial in our center (NCT04425226), pembrolizumab treatment needs to be stopped until>42 days before liver transplantation or unacceptable toxicity develops.

### The Prognostic Effect of Immune Cells on Survival

The density of Tumor-Infiltrating Lymphocytes (TILs) was found related to the survival rate of HCC (25). A high density of CD3+ and CD8+ T cells in both the interior and margin of HCC tumors was significantly associated with a low recurrence rate and prolonged RFS (26, 27). It was also correlated with PD-L1 staining, which was commonly used as a biomarker to evaluate the efficacy of PD-1 blockers. In other types of tumors, such as non-small cell lung cancer (NSCLC), high levels of CD8+TIL also had an excellent prognostic effect on survival (28). The expression of NK cells was also believed to be positively correlated with tumor cell apoptosis and negatively correlated with tumor proliferation (29). Therefore, in transplantation patients using ICIs, CD8+TIL and NK cell expression may be a marker for predicting better tumor prognosis.

## Conclusions

There are no guidelines or consensus on how and when to use ICIs in liver transplantation recipients due to safety concerns. The current research preliminarily proved that under close monitoring, the use of ICIs for organ transplant patients is feasible and effective. In the face of the possible trade-off between the risk of graft loss and the antitumor efficacy of ICIs, future research is needed in the future to clarify the risk factors that lead to rejection as early as possible, reduce the patient’s death risk, and improve the antitumor efficacy.

## Data Availability Statement

The original contributions presented in the study are included in the article/**Supplementary Material**. Further inquiries can be directed to the corresponding authors.

## Ethics Statement

The studies involving human participants were reviewed and approved by Ethical Committee of Renji Hospital. The patients/participants provided their written informed consent to participate in this study. Written informed consent was obtained from the individual(s) for the publication of any potentially identifiable images or data included in this article.

## Author Contributions 

HF and Z-yQ had full access to all of the data in the study and take responsibility for the integrity of the data and the accuracy of the data analysis. Concept and design: HF and QX. Acquisition, analysis, or interpretation of data: All authors. Drafting of the manuscript: Z-yQ and HF. Critical revision of the manuscript for important intellectual content: All authors. Data analysis: Z-yQ, Z-jZ and H-xW. Obtained funding: QX and HF. Administrative, technical, or material support: QX, J-jZ, X-sC, LX, and HT. Supervision: HF, QX, and J-jZ. All authors contributed to the article and approved the submitted version.

## Funding

This study was supported by the Funding Program from National Key Research and Development Project(2017YFC0908100), Shanghai Jiao Tong University (SJTU) Cross-disciplinary project (HF, YG2017QN54), Shanghai Natural Science Foundation (HF, 18ZR1424200), National Natural Science Foundation, China (HF, 81902388), Shanghai Medical Innovation Program (HF, 20Y11908900).

## Conflict of Interest

The authors declare that the research was conducted in the absence of any commercial or financial relationships that could be construed as a potential conflict of interest.

## Supplementary Material

Supplemental Figure 1 Postoperative immunosuppressive treatment and liver function of three representative patients in our center. **(A)** the medical treatment of patient 1 postoperatively **.(B, C)** the medical treatment of patient 2 and 3 postoperatively. EC-MPS, Mycophenolate Sodium; MP, methylprednisolon; Tac, tacrolimus; Pred, prednisone; SRL, sirolimus; ALT, AST(U/L); TB, Cr(μmol/L); IL-6, IL-8, IL-10 (pg/mL).

Supplemental Figure 2 HE staining of the specimens of the 3rd patient. **(A)** 40 times the microscope field of view of the HE stained liver biopsy. **(B, C)**, and **(D)** showed 100, 200, 400 times the microscope field of view. Pathological indicated an acute rejection (RAI=2+1+2 = 5), with mild expansion of the portal area, inflammatory cell reaction, cholangitis, and venous endothelitis.

## Abbreviations

AFP, alpha‐fetoprotein; ALT, alanine aminotransferase; AMR, Antibody-Mediated Rejection; AST, aspartate aminotransferase; CR, complete remission; CT, computed tomography; CMV, Cytomegalovirus; CTLA-4, cytotoxic T-lymphocyte- associated-4; LT, liver transplant; HCC, hepatocellular carcinoma; HBV infection, hepatitis B viral infection; ICU, Intensive care unit; IFN-γ/INFγ, interferon-gamma; IL2, interleukin 2; IL12p70, Interleukin-12, p70; ICIs, Immune checkpoint inhibitors; MRI, magnetic resonance imaging; MPNST, malignant peripheral nerve sheath tumor; NSCLC, non-small cell lung cancer; OLT, orthotopic liver transplantation; ORR, overall response rate; PR, partial remission; POD, postoperative day; PD-1, Programmed cell death 1; PD-L1, programmed death-ligand 1; PD, progressive disease; PIKVA-II, Protein induced by Vitamin K Absence or Antagonist-II; RAI, rejection activity index; TACE, transarterial chemotherapy; Th2, T helper cell type 2; TNFα, tumor necrosis factor alpha; TCMR, T Cell-Mediated Rejection.
