# Supplementary figures and images for: Neoadjuvant Programmed Cell Death 1 (PD-1) Inhibitor Treatment in Patients With Hepatocellular Carcinoma Before Liver Transplant: A Cohort Study and Literature Review
Source: Front Immunol. 2021 Jul 19;12:653437. doi: 10.3389/fimmu.2021.653437 (PMC8326904; doi:10.3389/fimmu.2021.653437)

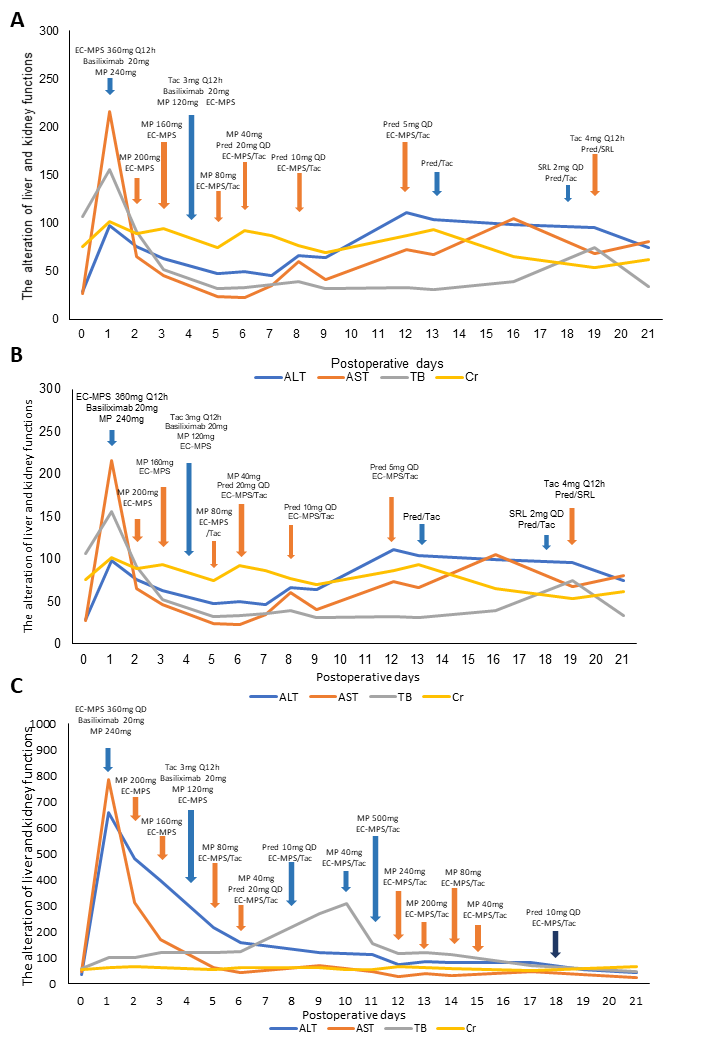

Supplement: Supplemental Figure 1 — Postoperative immunosuppressive treatment and liver function of three representative patients in our center. (A) the medical treatment of patient 1 postoperatively .(B, C) the medical treatment of patient 2 and 3 postoperatively. EC-MPS, Mycophenolate Sodium; MP, methylprednisolon; Tac, tacrolimus; Pred, prednisone; SRL, sirolimus; ALT, AST(U/L); TB, Cr(μmol/L); IL-6, IL-8, IL-10 (pg/mL). [file Image_1.tif]

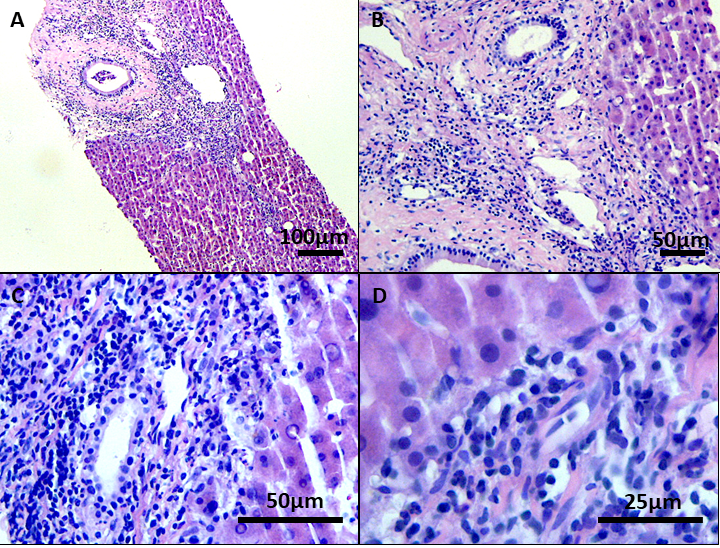

Supplement: Supplemental Figure 2 — HE staining of the specimens of the 3rd patient. (A) 40 times the microscope field of view of the HE stained liver biopsy. (B, C), and (D) showed 100, 200, 400 times the microscope field of view. Pathological indicated an acute rejection (RAI=2+1+2 = 5), with mild expansion of the portal area, inflammatory cell reaction, cholangitis, and venous endothelitis. [file Image_2.tif]
